# Cytotoxic and Anti-Inflammatory Activities of Dihydroisocoumarin and Xanthone Derivatives from *Garcinia picrorhiza*

**DOI:** 10.3390/molecules26216626

**Published:** 2021-11-01

**Authors:** Edwin R. Sukandar, Sutin Kaennakam, Pia Raab, Xuehong Nöst, Kitiya Rassamee, Rudolf Bauer, Pongpun Siripong, Taslim Ersam, Santi Tip-pyang, Warinthorn Chavasiri

**Affiliations:** 1 Center of Excellence in Natural Products Chemistry, Department of Chemistry, Faculty of Science, Chulalongkorn University, Bangkok 10330, Thailand; Edwin.R@chula.ac.th (E.R.S.); santi.ti@chula.ac.th (S.T.-p.); 2 Department of Agro-Industrial, Food, and Environmental Technology, Faculty of Applied Science, King Mongkut’s University of Technology North Bangkok (KMUTNB), Bangkok 10800, Thailand; n-s-k-@hotmail.com; 3 Department of Pharmacognosy, Institute of Pharmaceutical Sciences, University of Graz, Beethovenstraβe 8, 8010 Graz, Austria; pia.raab@uni-graz.at (P.R.); xuehong.noest@outlook.com (X.N.); rudolf.bauer@uni-graz.at (R.B.); 4 Natural Products Research Section, Research Division, National Cancer Institute, Bangkok 10400, Thailand; kalamae-nb@hotmail.com (K.R.); siripong_nci@yahoo.com (P.S.); 5 Natural Products and Synthesis Chemistry Research Laboratory, Department of Chemistry, Faculty of Science and Data Analytics, Institut Teknologi Sepuluh Nopember, Kampus ITS-Sukolilo, Surabaya 60111, Indonesia; paktichem@gmail.com

**Keywords:** xanthone, isocoumarin, Clusiaceae, *Garcinia picrorhiza*, cytotoxic, anti-inflammatory

## Abstract

*Garcinia picrorhiza*, a woody plant native to Sulawesi and Maluku Islands, Indonesia, has been traditionally used as a wound healing ointment. In our continuous search for bioactive compounds from this plant, 15 phenolic compounds were isolated from its stem bark, including a previously undescribed dihydroisocoumarin, 2′-hydroxyannulatomarin, and two undescribed furanoxanthones, gerontoxanthone C hydrate and 3′-hydroxycalothorexanthone. The structures of the new metabolites were elucidated on the basis of spectroscopic analysis, including 1D and 2D NMR and HRESIMS. Gerontoxanthone C hydrate possessed cytotoxicity against four cancer cells (KB, HeLa S3, MCF-7, and Hep G2) with IC_50_ values ranging from 5.6 to 7.5 µM. Investigation on the anti-inflammatory activities showed that 3′-hydroxycalothorexanthone inhibited NO production in RAW 264.7 and BV-2 cell lines with IC_50_ values of 16.4 and 13.8 µM, respectively, whereas only (−)-annulatomarin possessed inhibition activity on COX-2 enzyme over 10% at 20 µM. This work describes the presence of 3,4-dihydroisocoumarin structures with a phenyl ring substituent at C-3, which are reported the first time in genus *Garcinia*. These findings also suggest the potential of furanxanthone derivatives as cytotoxic and anti-inflammatory agents for further pharmacological studies.

## 1. Introduction

The genus *Garcinia*, one of the largest genera to the family Clusiaceae, is widespread in tropical rain forests with the center points are in Southeast Asia and Madagascar. This genus is pantropical and comprises a high level of species diversity with more than 250 species of evergreen, lactiferous, dioecious, and small shrubs to medium-sized trees [1,2]. Phytochemical analysis of genus *Garcinia* resulted in the isolation of structurally diverse secondary metabolites, especially phenolic compounds, such as xanthones, polyprenylated benzoylphloroglucinols, biphenyls, depsidones, and biflavonoids and many of which gained great attention because of their biological and pharmacological activities [3]. For example, a caged xanthone named gambogic acid (GA) from *Garcinia hanburyi*, has completed its phase IIa clinical trial in China for patients with lung, colon, and renal cancers. GA has been proved to induce apoptosis, reverse multidrug resistance of cancer cells, inhibit cell proliferation, and possess anti-angiogenic activities [4].

*Garcinia picrorhiza* Miq. is a woody plant that can be found in Maluku Islands, Indonesia. The roots and latex have been traditionally used as a wound healing ointment and natural energy drink [5,6,7]. Previously, our group reported eleven polyprenylated benzoylphloroglucinols (PPBPs), including eight derivatives (Picrorhizone A‒H) bearing a cyclobutane-containing side chain, with cytotoxic and anti-inflammatory activities. In particular, picrorhizone F was active against four human cancer cells (KB, HeLa S3, MCF-7, and Hep G2) with IC_50_ values in the range of 5.9‒9.4 µM, while picrorhizone H exhibited the highest COX-1 inhibitory activity (35.2 ± 9.6% inhibition) at 20 µM [8]. Encouraged by structurally diverse bioactive compounds from *Garcinia* species [8,9,10,11,12], we decided to revisit *G. picrorhiza* in part of a comprehensive phytochemical and biological investigation. Herein, we report the isolation, structural elucidation, and cytotoxic and anti-inflammatory effects of the phenolic compounds from the stem bark of *G. picrorhiza*.

## 2. Results and Discussion

### 2.1. Structural Elucidation of the Isolated Compounds

**Figure 1:**
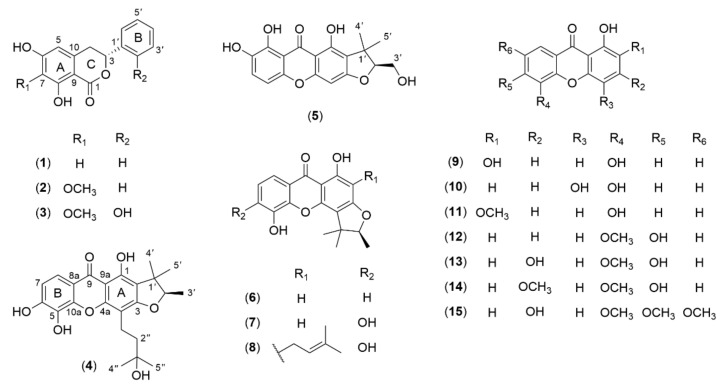
Isolated compounds (**1**‒**15**) from the stem bark of *Garcinia picrorhiza*.

The CH_2_Cl_2_-soluble fraction from the stem bark of *Garcinia picrorhiza* was subjected to a combination of chromatographic methods using silica gel, Sephadex LH-20, and chromatotron to give 15 compounds, including a new dihydroisocoumarin derivative, 2′-hydroxyannulatomarin (**3**), and two new furanoxanthones, gerontoxanthone C hydrate (**4**) and 3′-hydroxycalothorexanthone (**5**). The structures of the known compounds were determined as (−)-montroumarin (**1**) [13], (−)-annulatomarin (**2**) [14,15], pancixanthone B (**6**) [16], 2-deprenylrheediaxanthone B (**7**) [17], formoxanthone C (**8**) [18], 1,2,5-trihydroxyxanthone (**9**) [19], subelliptenone G (**10**) [20], 1,5-dihydroxy-2-methoxyxanthone (**11**) [21], 1,6-dihydroxy-5-methoxyxanthone (**12**) [22], 1,3,6-trihydroxy-5-methoxyxanthone (**13**) [23], 1,6-dihydroxy-3,5-dimethoxyxanthone (**14**) [24], and 1,3-dihydroxy-5,6,7-trimethoxyxanthone (**15**) [25] by comparison of their spectroscopic data with the literature (Figure 1).

**Figure 2:**
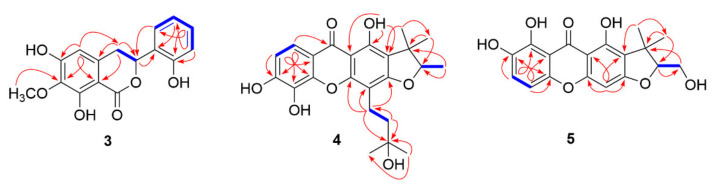
Key COSY (blue line) and HMBC (red arrow) correlation of compounds **3**‒**5**.

Compound **3** was obtained as a pale yellow amorphous solid with a molecular formula of C_16_H_14_O_6_ based on its molecular ion peak at *m*/*z* 325.0693 [M + Na]^+^ in HRESIMS, indicative of ten degrees of unsaturation. The IR spectrum of **3** indicated the presence of hydroxyl groups at 3187 cm^−1^ and aromatic moieties at 1630 and 1589 cm^−1^. The 1D NMR data of **3** were found to be similar to those of annulatomarin, a 3,4-dihydroisocoumarin derivative isolated from an herbaceous plant, *Hypericum annulatum* [14]. The typical carbonyl signal at *δ*_C_ 171.3 and the COSY cross-peak of H-3 [*δ*_H_ 5.93 (dd, *J* = 11.6, 4.0 Hz)]/H-4 [*δ*_H_ 3.18 (dd, *J* = 16.4, 11.6 Hz) and 3.13 (dd, *J* = 16.4, 4.0 Hz)] and HMBC correlations of H-3 to C-1′ (*δ*_C_ 126.2) and H-4 to C-5 (*δ*_C_ 107.3), C-9 (*δ*_C_ 102.7), and C-10 (*δ*_C_ 137.4) solidified the previous hypothesis (Table 1 and Figure 2). However, a principal difference was found in which four aromatic protons at *δ*_H_ 7.49 (d, *J* = 8.0, H-6′), 7.21 (t, *J* = 8.0, H-4′), 6.95 (d, *J* = 8.0, H-3′), 6.94 (t, *J* = 8.0, H-5′) were observed in the ^1^H-NMR spectrum of **3**, instead of five aromatic proton signals belonging to the phenyl ring of annulatomarin. The COSY experiment showed a contiguous spin system from H-3′ to H-6′ and HMBC spectrum exhibited correlations from H-3′ to C-2′ and C-5′, H-4′ to C-2′ and C-6′, and H-5′ to C-1′. The above 2D NMR analysis constructed an ortho-disubstituted benzene moiety at the B-ring of **3** as shown in Figure 2. Compound **3** was identified to be levorotary (${\left[\alpha \right]}_{D}^{20}\;$= ‒40.2) and showed a negative ECD Cotton effect (CE) at 276 nm followed by low-amplitude positive and negative CEs at 256 and 232 nm, respectively (See Supplementary Materials Figure S20). The two chiroptical data were in good agreement with previous results and thus implied that **3** favored 3*R* configuration [13,26,27]. The similar pattern of experimental ECD spectra of **1** and **2** with those of **3** indicated that they shared the same configuration at C-3 (Figure S20). It is worth noting that there was no clear evidence to prove the 3*R*-configuration of **2** in the previous report [15]. Hence, chiroptical data analysis was used in this work to assign the stereochemistry and compound **2** was defined to be (−)-annulatomarin.

Compound **4** was isolated as a pale yellow amorphous solid and its molecular formula was determined as C_23_H_26_O_7_ from the molecular ion peak at *m*/*z* 437.1663 [M + Na]^+^ in HRESIMS analysis, which in turn assigned 11 degrees of unsaturation. The ^1^H- and ^13^C- NMR spectra (Table 1) indicated **4** to feature a xanthone skeleton, especially the typical resonances for hydrogen-bonded hydroxy proton at *δ*_H_ 13.26 (s, OH-1) and carbonyl carbon at *δ*_C_ 182.0 [28]. Detailed analysis of 1D NMR data suggested that **4** had a scaffold similar to gerontoxanthone C [29], except that signals corresponding to a double bond of prenyl unit were missing in **4**. Instead, resonances for two methylenes at *δ*_H_ 2.91 (m, H-1″) and 1.81 (m, H-2″) and two methyls at *δ*_H_ 1.32 (each 3H, s, H-4″, and H-5″) indicated the hydration of the prenyl side chain and the COSY and HMBC spectra (Figure 2) further established the planar structure of **4**. The positive specific rotation (${\left[\alpha \right]}_{D}^{20}\;$= +35.7) and the ECD data (λ_max_ (Δɛ): 255 (‒5.0), 303 (+2.0), 330 (‒2.0) nm) of **4** (Figure S20) were in opposite signs with those of cudracuspixanthone B [30]. Therefore, the 2*′R* configuration of **4** was assigned.

Compound **5** was isolated as a pale yellow amorphous solid and the HRESIMS data showed a [M + H]^+^ peak at *m*/*z* 345.0971, indicative of a molecular formula C_18_H_16_O_7_. A comparison of the NMR data of **5** with those of calothorexanthone [31] showed that the difference was restricted to the trimethylfuran moiety in ring A. An oxymethylene proton at *δ*_H_ 3.91 (d, *J* = 5.6 Hz, H-3′) was observed in **5** instead of a methyl proton resonance as in the reference (Table 1). The COSY cross-peak of H-2′/H-3′ and HMBC correlations of H-3′ to C-1′ and C-2′ showed that the methyl group at C-2′ was oxidized to be a hydroxymethyl unit (Figure 2), which was supported by its HRESIMS analysis indicating one more oxygen atom than calothorexanthone. Unfortunately, the ECD spectrum of **5** could not be obtained due to the lack of sample for analysis and the positive optical rotation value (${\left[\alpha \right]}_{D}^{20}\;$= +22.9) was used to determine 2′*S* configuration in **5** [30].

Among the known metabolites, compounds **1**, **2**, **8**, **11**, and **15** were isolated for the first time from genus *Garcinia*. To the best of our knowledge, compounds **1**–**3** with the 3-phenyl-3,4-dihydroisocoumarin structure are the first occurrence in genus *Garcinia*, while earlier works reported its analogs containing an alkyl chain at C-3 instead of a phenyl ring, such as (‒)-mellein from *G. bancana* [32] and angelicoin B from *G. xanthochymus* [33]. Compounds **1** and **2** were previously obtained from the plant extracts of *Montrouziera sephaeroidea*, *Hypericum annulatum*, *Cratoxylum sumatranum* ssp. *neriifolium*, and some *Dioscorea* species [15,34,35,36]. This class of compounds is common metabolites in fungi, lichens, and bacteria, while their existence in higher plants is limited [37].

### 2.2. Cytotoxic Activity against Human Cancer Cell Lines

The cytotoxic activity of the isolated compounds was evaluated using the MTT viability assay [38] with doxorubicin as the positive control and the results are presented in Table 2. The active compounds having IC_50_ values lower than 30 µM against KB and HeLa S3 cell lines were further tested against the other three cancer cells (MCF-7, Hep G2, and HT-29). None of the dihydroisocoumarins **1**‒**3** possessed potent cytotoxicity with IC_50_ values over 30 µM. Among the group of xanthones, compound **4** was active against four cancer cells (KB, HeLa S3, MCF-7, and Hep G2) with IC_50_ values ranging from 5.6 to 7.5 µM. Our earlier studies [11,39] reported that furanoxanthones **7** and **8** also significantly inhibited the growth of KB, HeLa S3, MCF-7, and Hep G2 cancer cells with IC_50_ values less than 10 µM, while decreased cytotoxicity was observed for compound **6** with only one hydroxy group in the ring B of the xanthone skeleton. The hydration of the prenyl unit in **8** did not affect cytotoxic properties against the four cancer cells, as shown in schomburgone F [39]. These results suggested that trimethylfuran ring and ortho hydroxy unit might be required to enhance the cytotoxicity compared to the other isolated xanthones.

### 2.3. Nitric Oxide Inhibitory Activity

**Figure 3:**
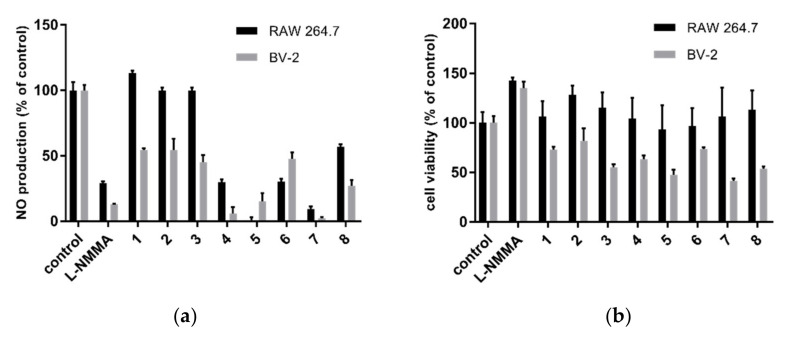
(**a**) NO production inhibitory effects and (**b**) cytotoxic properties of compounds **1**‒**8** at a final concentration of 50 µM in LPS-IFN-γ-induced RAW 264.7 macrophages and BV-2 microglial cells. L-NMMA was used as the positive control at 100 µM. The NO inhibition of control group (LPS-IFN-γ-induced cells with 1% DMSO) was set as 100% (*n* = 6).

Compounds **1**–**8** were screened for their inhibitory effects on nitric oxide production in LPS-IFN-γ activated RAW 264.7 macrophages and BV-2 microglial cells at a final concentration of 50 µM. The preliminary results (Figure 3) indicated that dihydroisocoumarins **1**–**3** were weak to inactive on NO inhibitory activity, whereas **4**, **5**, and **7** suppressed NO production over 70% in both cell lines. A deprenylated furanoxanthone structure with ortho hydroxy group in ring B as in **7** might be important to enhance the inhibitory effect when compared to **6** and **8**. None of the tested compounds showed obvious cytotoxicity towards RAW 264.7 cells (cell viability >90%), while the survival of BV-2 cells was reduced with cell viability in the range of 41.8–81.5% after compound treatment at 50 µM (Figure 3). IC_50_ values of the three active compounds are listed in Table 3. Compound **5** possessed the strongest inhibitory activity against NO production in RAW 264.7 and BV-2 cells with IC_50_ values of 16.4 and 13.8 µM, respectively. Compounds **4** and **7** moderately inhibited NO production in RAW 264.7 cells, whereas their anti-inflammatory effect in BV-2 cells might be masked cytotoxicity (IC_50_ values of their NO inhibition were only 1.4 and 1.1-fold lower than the cytotoxic activity).

### 2.4. Cyclooxygenase (COX) Inhibitory Activity

The anti-inflammatory effects of the isolated compounds were also screened on COX-1 and COX-2 enzymes inhibition at a final concentration of 20 µM. The results are given in Table 4. Only compound **2** exhibited activity against COX-2 with 10.4% inhibition. All furanoxanthones (**4**‒**8**), except **5** with oxygenated trimethylfuran moiety, showed COX-1 inhibition higher than 10% with the new compound **4** possessing the strongest activity (32.4 ± 7.9% inhibition). Removal of the hydroxy group at C-2 in **9** reduced the COX-1 inhibition when compared with **10** and **11**, while introducing a methoxy group at C-3 in **14** increased the inhibitory effect compared to **12** and **13**.

## 3. Materials and Methods

### 3.1. General Experimental Procedures

Optical rotations were measured on a JASCO P-1010 polarimeter (JASCO, Easton, MD, USA). The experimental ECD data were recorded on a JASCO J-815 circular dichroism spectrometer (JASCO, Easton, MD, USA). The IR data were obtained with a Nicolet 6700 FT-IR spectrometer using an ATR technique (Thermo Fisher Scientific, Waltham, MA, USA). The NMR spectra were acquired on a Bruker 400 AVANCE spectrometer in acetone-*d*_6_ (Merck, Darmstadt, Germany). The HRMS spectra were recorded using a Bruker MICROTOF model mass spectrometer (Bruker, Billerica, MA, USA) and Dionex Ultimate 3000 HPLC system hyphenated with a QExactive Hybrid Quadrupole Orbitrap MS (Thermo Fisher Scientific). Silica gel 70–230 mesh (Merck) and Sephadex LH-20 (GE Healthcare Bio-Sciences AB, Uppsala, Sweden) were used for column chromatography. Radial chromatography (Chromatotron model 7924 T, Harrison Research, Palo Alto, CA, USA) was carried out with silica gel 60 GF254 containing gypsum (Merck).

### 3.2. Plant Material

The stem bark of *G. picrorhiza* Miq. (Clusiaceae) was collected from Bogor Botanical Garden, Bogor, Indonesia (6°35′51″ S 106°47′55″ E) in July 2006. The plant material was identified by Dr. Rismita Sari. A voucher specimen (No. VI.A.26) was deposited at Bogor Botanical Garden, Indonesia.

### 3.3. Extraction and Isolation

The *G. picrorhiza* stem bark (3.0 kg) was extracted with MeOH (15 L each/3 days). The crude extract (92.0 g) was partitioned with CH_2_Cl_2_ and EtOAc solvents to yield two organic fractions. The CH_2_Cl_2_-soluble fraction (54.1 g) was separated by silica gel column chromatography with hexanes/EtOAc (95:5–0:100) to afford fractions A‒R, following the previous procedure [8]. Fraction F (1.1 g) was chromatographed on a Sephadex LH-20 column with CH_2_Cl_2_/MeOH (1:1) to obtain subfractions F1–F4. Subfraction F3 (52.7 mg) was separated by a Chromatotron with eluent hexanes/CH_2_Cl_2_ (20:80) to yield compounds **10** (3.0 mg) and **12** (3.3 mg). Subfraction F4 (106.0 mg) was purified on a Chromatotron with an eluent system of hexanes/CH_2_Cl_2_ (40:60) to yield compounds **1** (6.4 mg), **2** (2.9 mg), **6** (3.8 mg), and **8** (3.6 mg). Fraction J (556.0 mg) was subjected to a Sephadex LH-20 column with CH_2_Cl_2_/MeOH (1:1) to yield subfractions J1‒J3. Purification of subfraction J2 (68.8 mg) using a Chromatotron with eluent hexanes/chloroform (60:40) yielded compounds **11** (4.2 mg) and **14** (3.5 mg). Fraction K (1.3 g) was chromatographed on a Sephadex LH-20 column eluted with CH_2_Cl_2_/MeOH (1:1) to obtain subfractions K1–K4. Subfraction K3 (408.1 mg) was separated using a Chromatotron with hexanes/chloroform (20:80‒0:100) followed by chloroform/MeOH (20:1) to afford compounds **3** (4.8 mg), **5** (1.0 mg), **7** (2.1 mg), **9** (4.2 mg), and **15** (1.1 mg). Compound **4** (4.0 mg) was isolated from fraction M (80.5 mg) through a separation using a Sephadex LH-20 column eluted with CH_2_Cl_2_/MeOH (1:1), while the same method was also applied to fraction O (79.2 mg) to yield compound **13** (5.6 mg).

2′-hydroxyannulatomarin (**3**). Pale yellow, amorphous solid; ${\left[\alpha \right]}_{D}^{20}\;$= ‒40.2 (*c* 0.05, MeOH); ECD λ_max_ (*c* 0.05, MeOH) nm (log ɛ): 276 (‒4.8), 256 (+1.0), 232 (‒2.0); IR (ATR) cm^−1^: 3187, 1630, 1589, 1507, 1256, 1096; ^1^H- (400 MHz, acetone-*d*_6_) and ^13^C-NMR (100 MHz, acetone-*d*_6_) spectroscopic data, see Table 1; HRESIMS *m*/*z* 325.0693 [M + Na]^+^ (calcd. for C_16_H_14_O_6_Na: 325.0688).

Gerontoxanthone C hydrate (**4**). Pale yellow, amorphous solid; ${\left[\alpha \right]}_{D}^{20}\;$= +35.7 (c 0.05, MeOH); ECD *λ*_max_ (*c* 0.05, MeOH) nm (log ɛ): 330 (‒2.0), 303 (+2.0), 255 (‒5.0), 236 (+0.9), 214 (‒7.4); ^1^H- (400 MHz, acetone-*d*_6_) and ^13^C-NMR (100 MHz, acetone-*d*_6_) spectroscopic data, see Table 1; HRESIMS *m*/*z* 437.1663 [M + Na]^+^ (calcd. for C_23_H_26_O_7_Na: 437.1576).

3′-hydroxycalothorexanthone (**5**). Pale yellow, amorphous solid; ${\left[\alpha \right]}_{D}^{20}\;$= +22.9 (*c* 0.05, MeOH); ^1^H- (400 MHz, acetone-*d*_6_) and ^13^C-NMR (100 MHz, acetone-*d*_6_) spectroscopic data, see Table 1; HRESIMS *m*/*z* 345.0971 [M + H]^+^ (calcd. for C_18_H_17_O_7_: 345.0974).

### 3.4. Cytotoxic Activity Assay

The MTT colorimetric method was performed to evaluate cytotoxic activity of the isolated compounds against human epidermoid carcinoma (KB; ATCC CCL17), human cervical carcinoma (HeLa S3; ATCC CCL2-2), human colon adenocarcinoma (HT-29; ATCC HTB-38), human breast adenocarcinoma (MCF-7; ATCC HTB-22), and human hepatocellular carcinoma (Hep G2; ATCC HB-8065) cell lines according to the previous method [38]. The cancer cells were cultured in MEM containing 10% fetal bovine serum in the presence of 100 U/mL penicillin and 100 μg/mL streptomycin sulphate (Gibco, Rockville, MD, USA), seeded in a 96-well plate (3000 cells/well), and pre-incubated at 37 °C for 24 h in a 5% CO_2_ humidified atmosphere. The tested compounds with serial concentrations (0.3–100 μM) were added and incubated for a further 72 h in the same condition. At the end of treatment, 20 µL of MTT solution (5 mg/mL in PBS, Sigma, St. Louis, MI, USA) was added to each well and further incubated for 3 h. After centrifugation at 1400 rpm for 5 min at 4 °C, the supernatant was decanted and DMSO (100 µL/well) was added to allow formazan product solubilization, which was subsequently measured by a microplate reader (Tecan Trading AG, Switzerland) at wavelength 550 nm. Control cells were treated with 0.1% DMSO. Doxorubicin (tested concentrations: 0.01–3.0 μM, Sigma) was used as the positive control. The results are expressed as the mean values of three replicates for three independent experiments. IC_50_ values were determined by graphical analysis using SigmaPlot 10 (Systat Software Inc., San Jose, CA, USA), obtained by plotting the cell viability percentage versus sample concentration.

### 3.5. NO Production Inhibition Assay

The iNOS inhibitory activity of the isolated compounds and respective cytotoxicity was determined using a previously described protocol [40]. Briefly, BV-2 mouse microglial (25,000 cells/well) and RAW 264.7 mouse macrophage (100,000 cells/well) cell lines were seeded into 96-well plates in high glucose Dulbecco’s Modified Eagle’s Medium (DMEM, Gibco) containing 4 mM L-glutamine, 10% FBS, 2% HEPES, 1% penicillin/streptomycin, and 0.5% amphotericin B. After incubation for 24 h at 37 °C in a 5% CO_2_ humidified atmosphere, the cells were stimulated with 0.05% LPS (Sigma) and 0.025% interferon-γ (Roche Diagnostics, Mannheim, Germany) and treated with different concentrations (5–100 μM) of the tested compounds. The level of NO production in cell culture supernatants was determined using Griess reagent (Sigma) by photocolorimetric analysis after 16 h of incubation, using a Hidex Sense Microplate Reader (Hidex, Turku, Finland). Fifty microliters of XTT reagent (Sigma, Kit II) was then added into the same plates for cell viability measurement after 60 min incubation. L-NMMA (Alexis, Grünberg, Germany) 100 µM was used as the positive control, while 1% DMSO and digitonin (Roth, Karlsruhe, Germany) 400 µM were used as the vehicle and negative control, respectively. The assays were conducted in triplicates and repeated in two independent experiments. IC_50_ values were determined by graphical analysis using GraphPad Prism 7 (GraphPad Software, La Jolla, CA, USA).

### 3.6. COX Enzymes Inhibition Assay

Purified PGHS-1 from ram seminal vesicles for COX-1 and human recombinant PGHS-2 for COX-2 (Cayman Chemical Co., Ann Arbor, MI, USA) were used for the COX inhibition assays. Experiments were performed in a 96-well plate format, as previously described [40,41]. Briefly, the incubation mixture contained 180 µL of 0.1 M TRIS/HCl-buffer pH 8.0 (Roth, Karlsruhe, Germany), 50 µM Na_2_EDTA (only for COX-2, Titriplex III, Merck, Darmstadt, Germany), 18 mM l-epinepherine bitartrate (Fluka, Buchs, Switzerland), 5 µM porcine hematin (MP Biomedicals LLC, Solon, OH, USA), and COX-1 or COX-2 enzymes (0.2 U/well). Ten microliters of tested compounds (dissolved in DMSO with a final concentration of 20 µM) was added and the mixture was pre-incubated for 5 min at room temperature. Thereafter, 10 µL of 5 µM arachidonic acid (Ann Arbor, Michigan USA) was added to the mixture and incubated for 20 min at 37 °C. The reaction was subsequently stopped by adding 10 µL of 10% (*v*/*v*) formic acid. The concentration of PGE_2_ generated in the reaction was quantitatively measured by a competitive PGE_2_ ELISA kit (Enzo Life Sciences Inc., Farmingdale, NY, USA) according to the manufacturer’s protocol and previous procedure [40]. The absorbance at 405 nm was measured using a Hidex Sense Microplate Reader (Hidex, Turku, Finland). Indomethacin (1.25 µM, dissolved in ethanol; MP Biomedicals, Solon, OH, USA) and celecoxib (8.8 µM, dissolved in DMSO; Sigma) were used as a positive control for COX-1 and COX-2, respectively. The assays were performed in duplicates and repeated in two independent experiments.

## 4. Conclusions

Phytochemical investigation of the stem bark of *G. picrorhiza* led to the isolation of 15 dihydroisocoumarins and xanthones, including three new analogs. The presence of dihydroisocoumarin structures with a phenyl moiety at C-3 is expected to enrich information about the chemical diversity of the genus *Garcinia*. This work also indicates that furanoxanthone structures are promising candidates and could be used as a template for discovering potential anticancer and anti-inflammatory agents.

## Figures and Tables

**Table 1:** ^1^H- (400 MHz) and ^13^C- (100 MHz) NMR spectroscopic data of compounds **3**‒**5** recorded in acetone-*d*_6_ (*δ* in ppm).

Position	3		Position	4		5	
	*δ*_H_ (*J* in Hz)	*δ*_C_		*δ*_H_ (*J* in Hz)	*δ*_C_	*δ*_H_ (*J* in Hz)	*δ*_C_
1		171.3	1		157.5		159.5
O-2			2		116.9		117.9
3	5.93, dd (11.6, 4.0)	77.0	3		164.5		168.2
4a	3.13, dd (16.4, 4.0)	34.1	4		104.6	6.38, s	90.4
4b	3.18, dd (16.4, 11.6)		4a		155.8		159.5
5	6.42, s	107.3	10a		146.9		149.8
6		157.7	5		133.4	6.90, d (8.8)	107.0
7		134.8	6		151.9	7.33, d (8.8)	124.6
8		157.7	7	6.96, d (8.4)	113.9		141.4
9		102.7	8	7.61, d (8.4)	117.3		148.0
10		137.4	8a		114.7		108.3
1′		126.2	9		182.0		185.5
2′		155.0	9a		104.4		103.4
3′	6.95, d (8.0)	116.4	1′		44.6		43.6
4′	7.21, t (8.0)	130.4	2′	4.53, q (6.4)	91.5	4.51, t (5.6)	96.0
5′	6.94, t (8.0)	120.8	3′	1.41, d (6.4)	14.8	3.91, d (5.6)	61.4
6′	7.49, d (8.0)	127.8	4′	1.24, s	21.1	1.38, s	20.6
OH-8	11.44, brs		5′	1.49, s	25.7	1.57, s	26.8
OCH_3_-7	3.84, s	60.8	1″	2.91, m	18.3		
			2″	1.81, m	42.9		
			3″		71.8		
			4″	1.32, s	29.7		
			5″	1.32, s	29.7		
			OH-1	13.26, s			

**Table 2:** Cytotoxic activity of the isolated compounds against five human cancer cell lines.

Compound	IC_50_ ± SEM (µM) ^a^				
	KB	HeLa S3	MCF-7	Hep G2	HT-29
**4**	7.5 ± 0.8	5.6 ± 0.1	5.7 ± 0.3	6.3 ± 0.6	20.3 ± 0.6
**6** ^b^	12.1 ± 0.1	20.7 ± 0.6	15.6 ± 0.3	22.8 ± 0.4	inactive
**7** ^b^	5.1 ± 0.4	6.0 ± 0.5	6.5 ± 0.2	10.0 ± 0.2	inactive
**8** ^b^	0.2 ± 0.1	0.3 ± 0.1	4.9 ± 0.4	3.8 ± 0.5	21.9 ± 1.2
**13**	11.4 ± 1.7	15.2 ± 1.3	NT	NT	NT
Doxorubicin ^c^	0.02 ± 0.01	0.15 ± 0.02	1.29 ± 0.02	1.00 ± 0.17	0.59 ± 0.03

^a^ Results are expressed as the means ± SEM of three replicates. ^b^ The cytotoxic results based on our previous work [11,39]. ^c^ Doxorubicin was used as the positive control. Note: NT= not tested, IC_50_ ≤ 10 µM = good cytotoxicity, 10 µM < IC_50_ ≤ 30 µM = weak cytotoxicity. The other compounds were inactive (IC_50_ > 30 µM).

**Table 3:** IC_50_ values (µM) ^d^ of compounds **4**, **5**, and **7** on inhibition of NO production in LPS-IFN-γ-induced RAW 264.7 macrophages and BV-2 microglial cells.

Compound	RAW 264.7		BV-2	
	iNOS	Cytotoxicity	iNOS	Cytotoxicity
**4**	84.3 ± 3.5	>200	20.0 ± 4.0	27.7 ± 6.4
**5**	16.4 ± 4.5	>200	13.8 ± 1.6	74.7 ± 2.1
**7**	45.6 ± 6.5	85.6 ± 6.9	28.7 ± 2.3	31.7 ± 4.7

^d^ Results are expressed as the means ± SEM (*n* = 6) of two independent experiments. L-NMMA at 100 µM was used as the positive control with inhibition of 64.1 ± 4.1% in RAW 264.7 and 44.1 ± 5.2% in BV-2 cell lines.

**Table 4:** Inhibitory activity ^e^ of the isolated compounds against COX enzymes.

Compound	% Inhibition at 20 µM	
	COX-1	COX-2
**2**	<10	10.4 ± 5.0
**3**	24.2 ± 12.3	<10
**4**	32.4 ± 7.9	<10
**6**	31.2 ± 21.7	<10
**7**	15.4 ± 7.5	<10
**8**	24.0 ± 15.0	<10
**9**	22.6 ± 2.2	<10
**14**	18.3 ± 12.1	<10
Indometacin ^f^	78.4 ± 4.1	NT
Celecoxib ^g^	NT	83.5 ± 4.8

^e^ Results are expressed as the means ± SD (*n* = 4) of two independent experiments. ^f^ Indometacin was used as the positive control for COX-1 at 1.25 µM. ^g^ Celecoxib was used as the positive control for COX-2 at 8.8 µM. The other compounds showed no activity against COXs with inhibition lower than 10%. NT = not tested.

## Data Availability

The supporting information can be found in the Supplementary Materials.

## Supplementary Materials

The following are available online, HRESIMS, 1D and 2D NMR spectra of **3**‒**5**, and experimental ECD spectra of **1**‒**4**.

## Sample Availability

Samples of the compounds are not available from the authors.
