# Supplementary material for: Cytotoxic and Anti-Inflammatory Activities of Dihydroisocoumarin and Xanthone Derivatives from Garcinia picrorhiza
Source: Molecules. 2021 Nov 1;26(21):6626. doi: 10.3390/molecules26216626 (PMC8587515; doi:10.3390/molecules26216626)
Supplement: Supplementary file 1 [file molecules-26-06626-s001.zip › molecules-1390066-supplementary.pdf]

SUPPORTING INFORMATION

Cytotoxic and anti-inflammatory activities of dihydroisocoumarin and xanthone derivatives from *Garcinia picrorhiza*

Edwin R. Sukandar[1], Sutin Kaennakam[2], Pia Raab[3], Xuehong Nöst[3], Kitiya Rassamee[4], Rudolf Bauer[3], Pongpun Siripong[4], Taslim Ersam[5], Santi Tip-pyang[1], and Warinthorn Chavasiri [1,*]

[1]Center of Excellence in Natural Products Chemistry, Department of Chemistry, Faculty of Science, Chulalongkorn University, Bangkok 10330, Thailand

[2]Department of Agro-Industrial, Food, and Environmental Technology, Faculty of Applied Science, King Mongkut's University of Technology North Bangkok (KMUTNB), Bangkok 10800, Thailand

[3]Institute of Pharmaceutical Sciences, Section of Pharmacognosy, University of Graz, Beethovenstraβe 8, 8010 Graz, Austria

[4]Natural Products Research Section, Research Division, National Cancer Institute, Bangkok 10400, Thailand

[5]Natural Products and Synthesis Chemistry Research Laboratory, Department of Chemistry, Faculty of Science and Data Analytics, Institut Teknologi Sepuluh Nopember, Kampus ITS-Sukolilo, Surabaya 60111, Indonesia

***Corresponding authors**

Assist. Prof. Dr. Warinthorn Chavasiri. Tel.: +662 218 762. E-mail address: warinthorn.c@chula.ac.th

Table of Contents

Figure S1. ^{1}H NMR spectrum of 2′-hydroxyannulatomarin (**3**) in acetone-d_6

Figure S2. ^{13}C NMR spectrum of 2′-hydroxyannulatomarin (**3**) in acetone-d_6

Figure S3. COSY spectrum of 2′-hydroxyannulatomarin (**3**) in acetone-d_6

Figure S4. HSQC spectrum of 2′-hydroxyannulatomarin (**3**) in acetone-d_6

Figure S5. HMBC spectrum of 2′-hydroxyannulatomarin (**3**) in acetone-d_6

Figure S6. HRESIMS spectrum of 2′-hydroxyannulatomarin (**3**) in MeOH

Figure S7. IR spectrum of 2′-hydroxyannulatomarin (**3**)

Figure S8. ^{1}H NMR spectrum of gerontoxanthone C hydrate (**4**) in acetone-d_6

Figure S9. ^{13}C NMR spectrum of gerontoxanthone C hydrate (**4**) in acetone-d_6

Figure S10. COSY spectrum of gerontoxanthone C hydrate (**4**) in acetone-d_6

Figure S11. HSQC spectrum of gerontoxanthone C hydrate (**4**) in acetone-d_6

Figure S12. HMBC spectrum of gerontoxanthone C hydrate (**4**) in acetone-d_6

Figure S13. HRESIMS spectrum of gerontoxanthone C hydrate (**4**) in MeOH

Figure S14. ^{1}H NMR spectrum of 3′-hydroxycalothorexanthone (**5**) in acetone-d_6

Figure S15. ^{13}C NMR spectrum of 3′-hydroxycalothorexanthone (**5**) in acetone-d_6

Figure S16. COSY spectrum of 3′-hydroxycalothorexanthone (**5**) in acetone-d_6

Figure S17. HSQC spectrum of 3′-hydroxycalothorexanthone (**5**) in acetone-d_6

Figure S18. HMBC spectrum of 3′-hydroxycalothorexanthone (**5**) in acetone-d_6

Figure S19. HRESIMS spectrum of 3′-hydroxycalothorexanthone (**5**) in MeOH

Figure S20. Experimental ECD spectra of **1–4** in MeOH

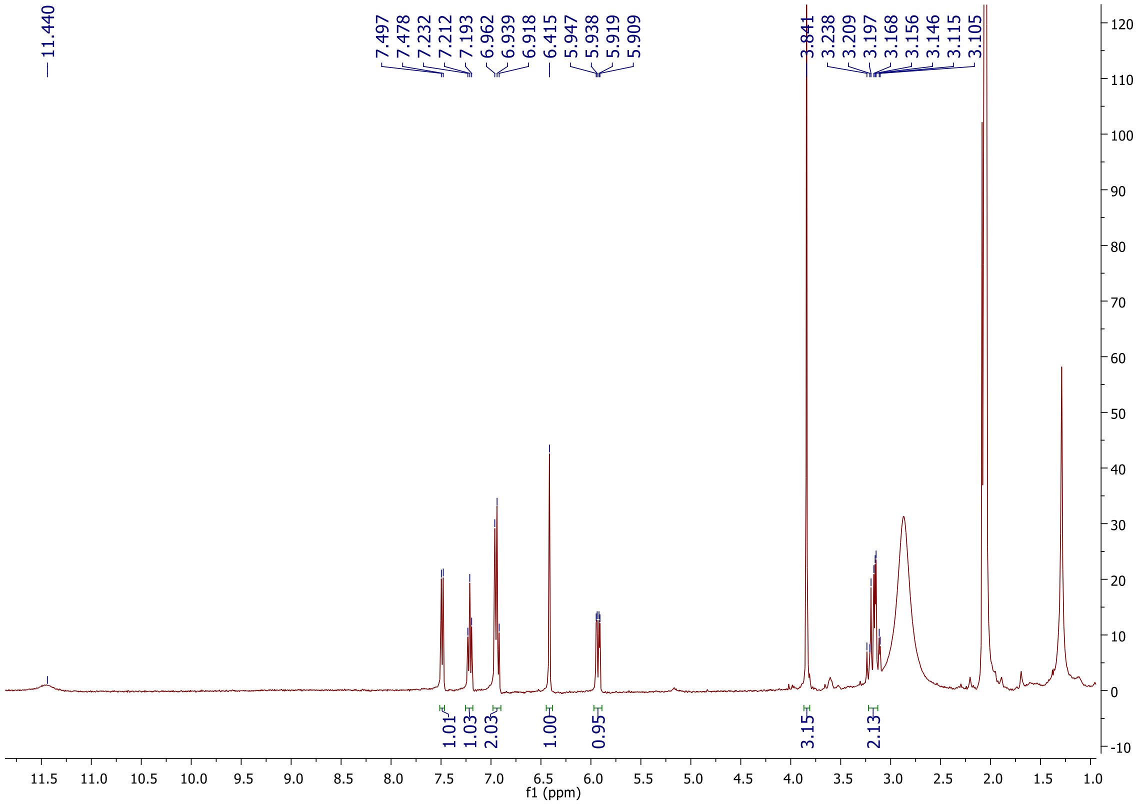

Figure S1. ^{1}H NMR spectrum of 2′-hydroxyannulatomarin (**3**) in acetone-d_6

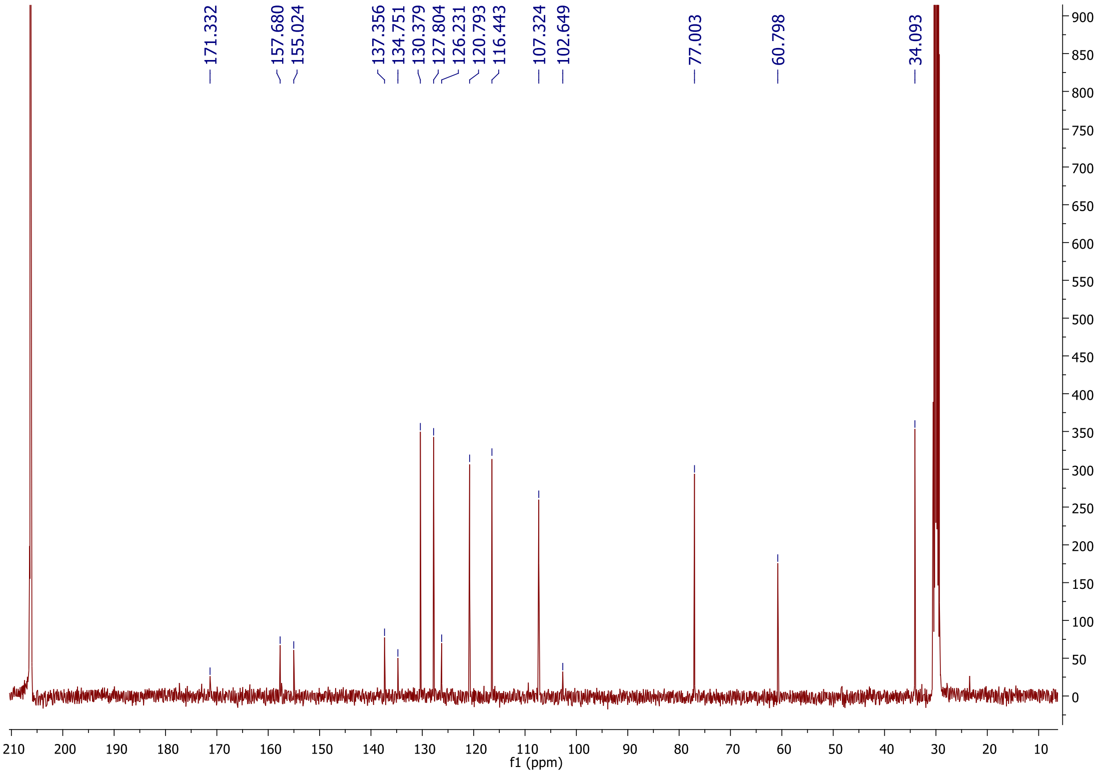

Figure S2. ^{13}C NMR spectrum of 2′-hydroxyannulatomarin (**3**) in acetone-d_6

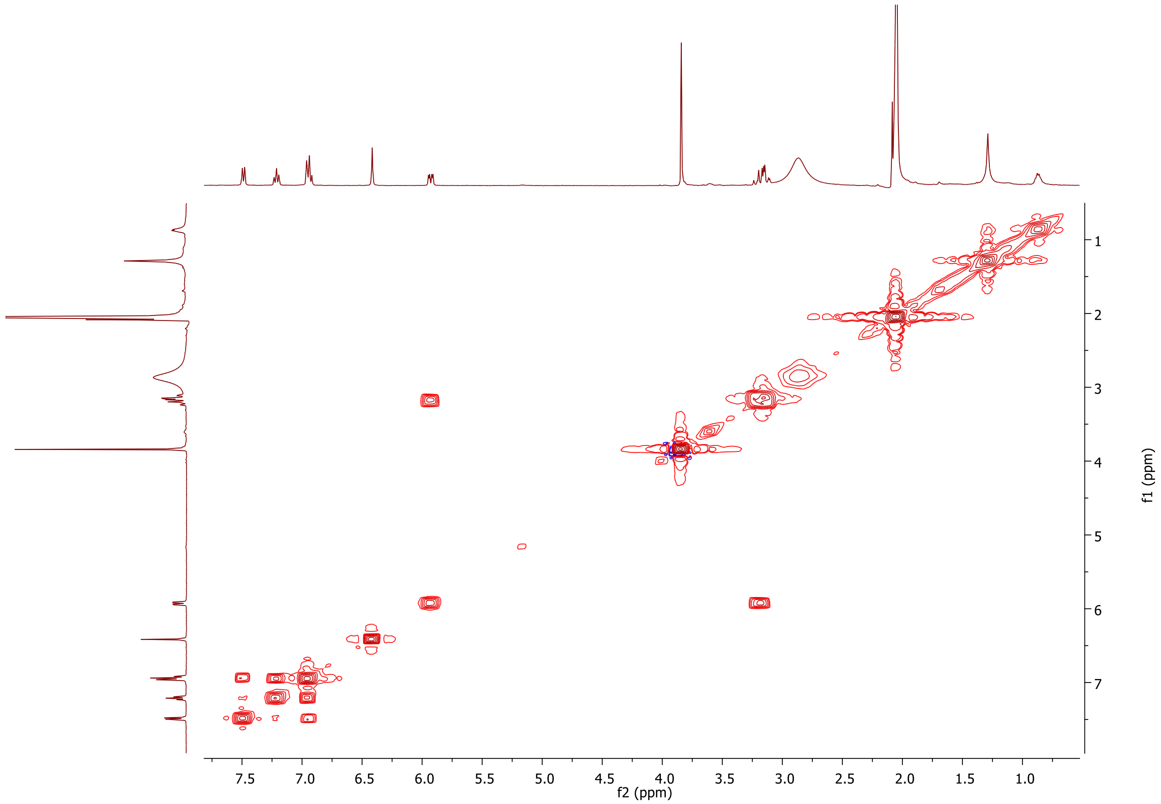

Figure S3. COSY spectrum of 2′-hydroxyannulatomarin (**3**) in acetone-d_6

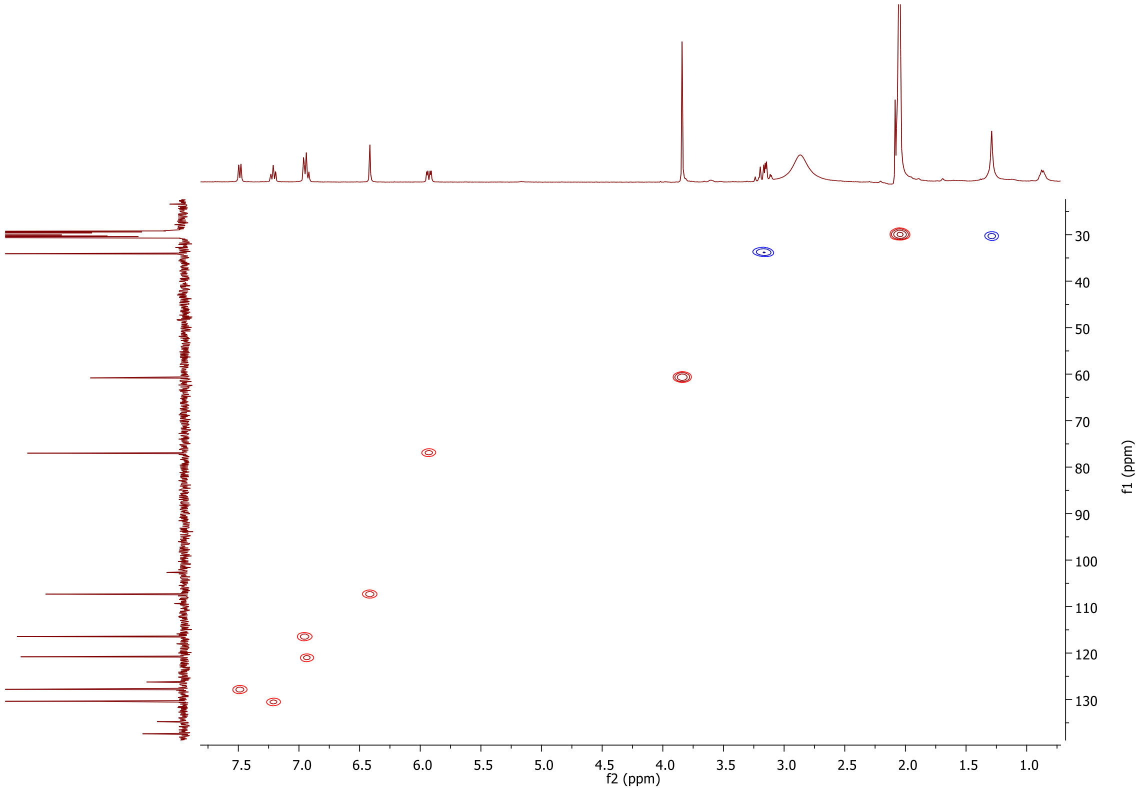

Figure S4. HSQC spectrum of 2′-hydroxyannulatomarin (**3**) in acetone-d_6

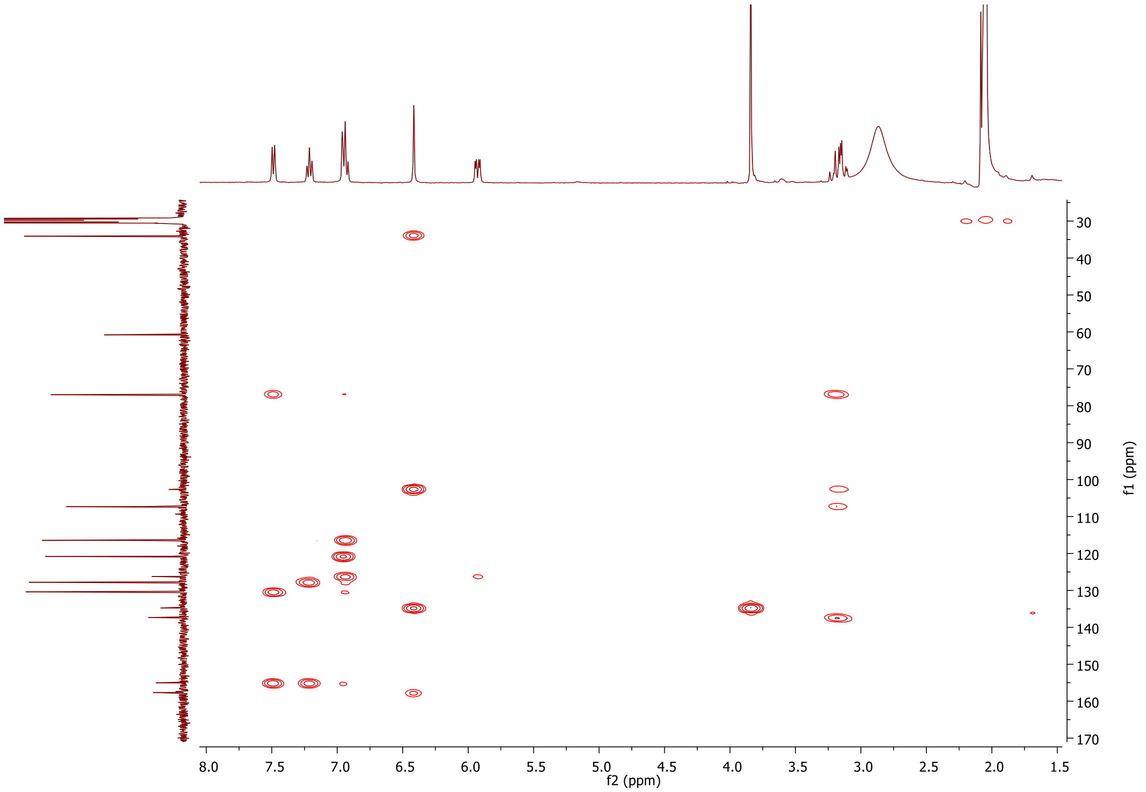

Figure S5. HMBC spectrum of 2′-hydroxyannulatomarin (**3**) in acetone-d_6

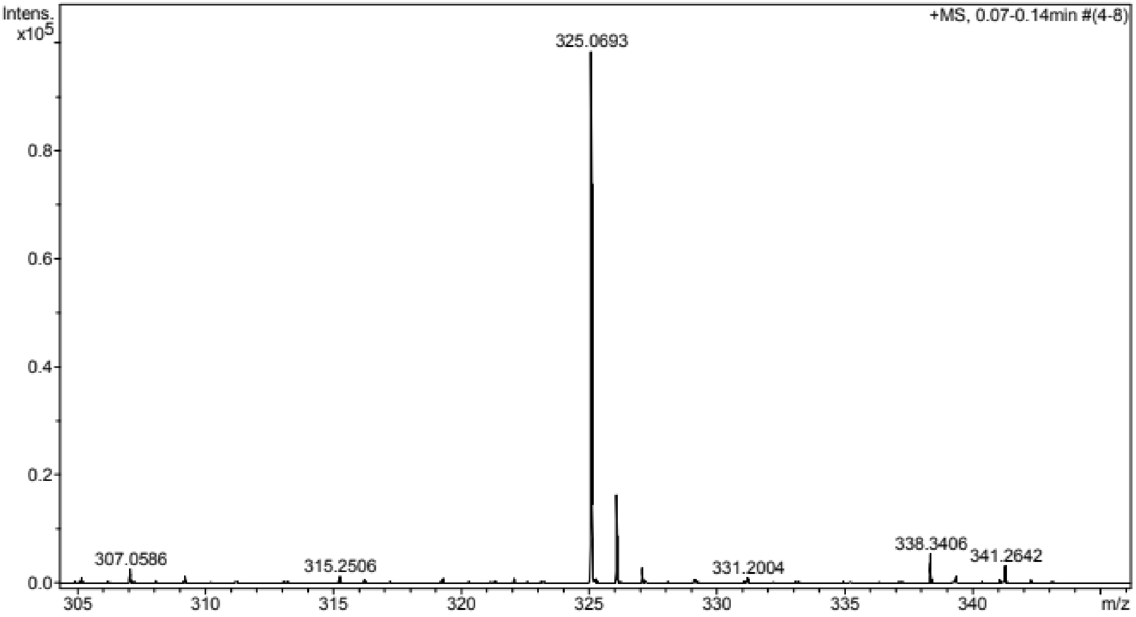

Figure S6. HRESIMS spectrum of 2′-hydroxyannulatomarin (**3**) in MeOH

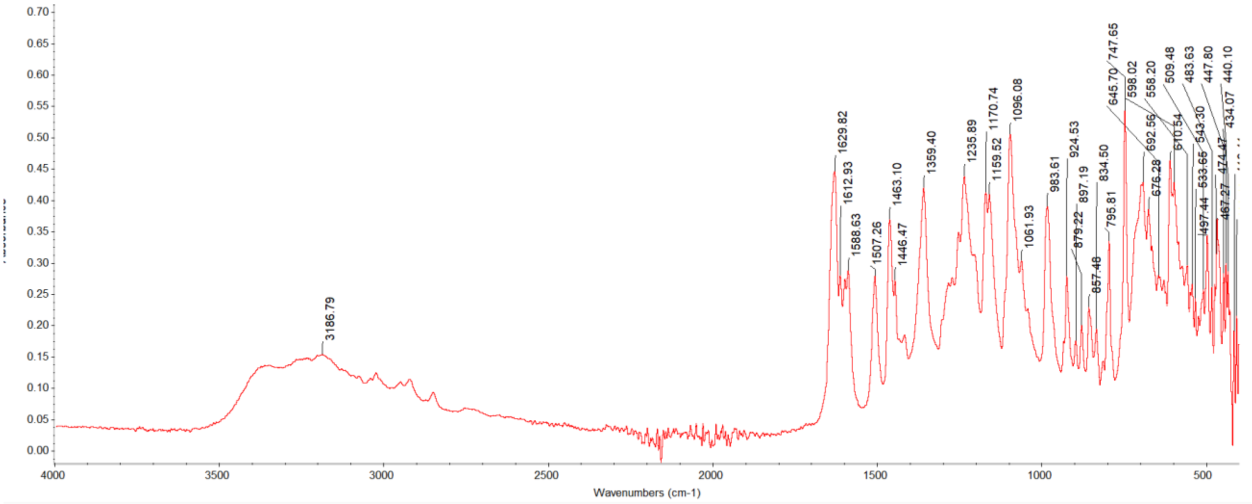

Figure S7. IR spectrum of 2′-hydroxyannulatomarin (**3**)

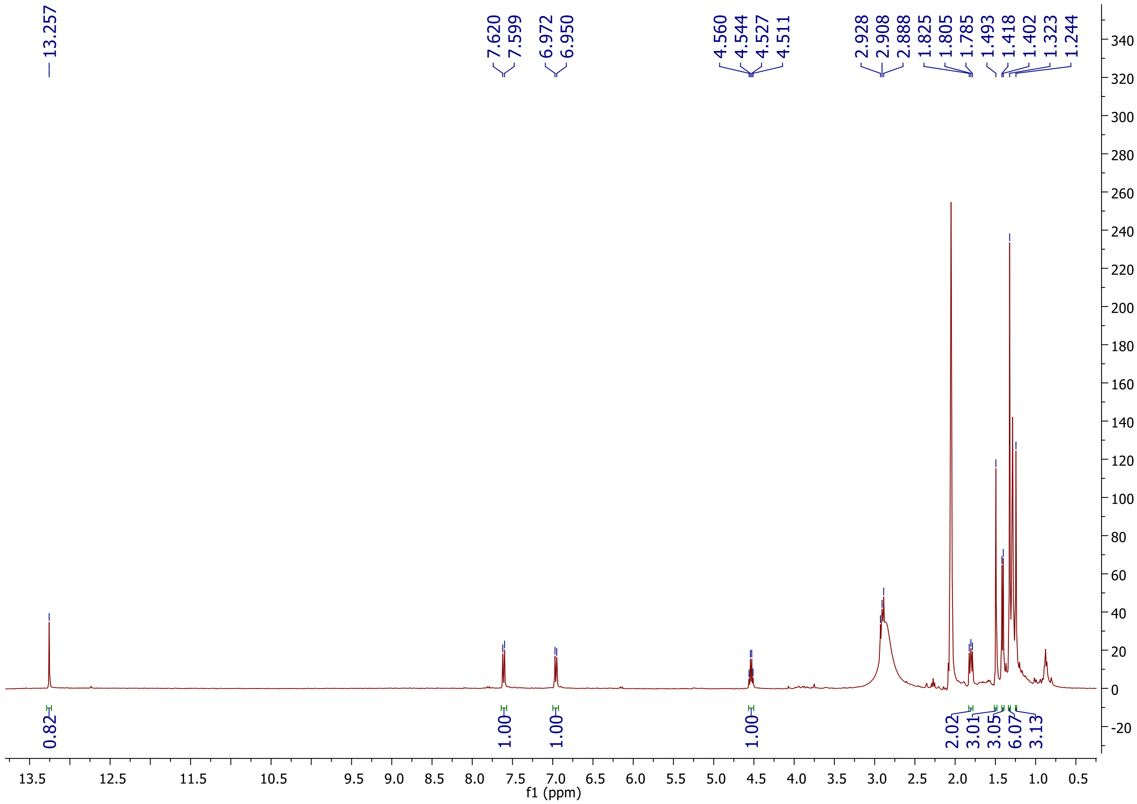

Figure S8. ^{1}H NMR spectrum of gerontoxanthone C hydrate (**4**) in acetone-d_6

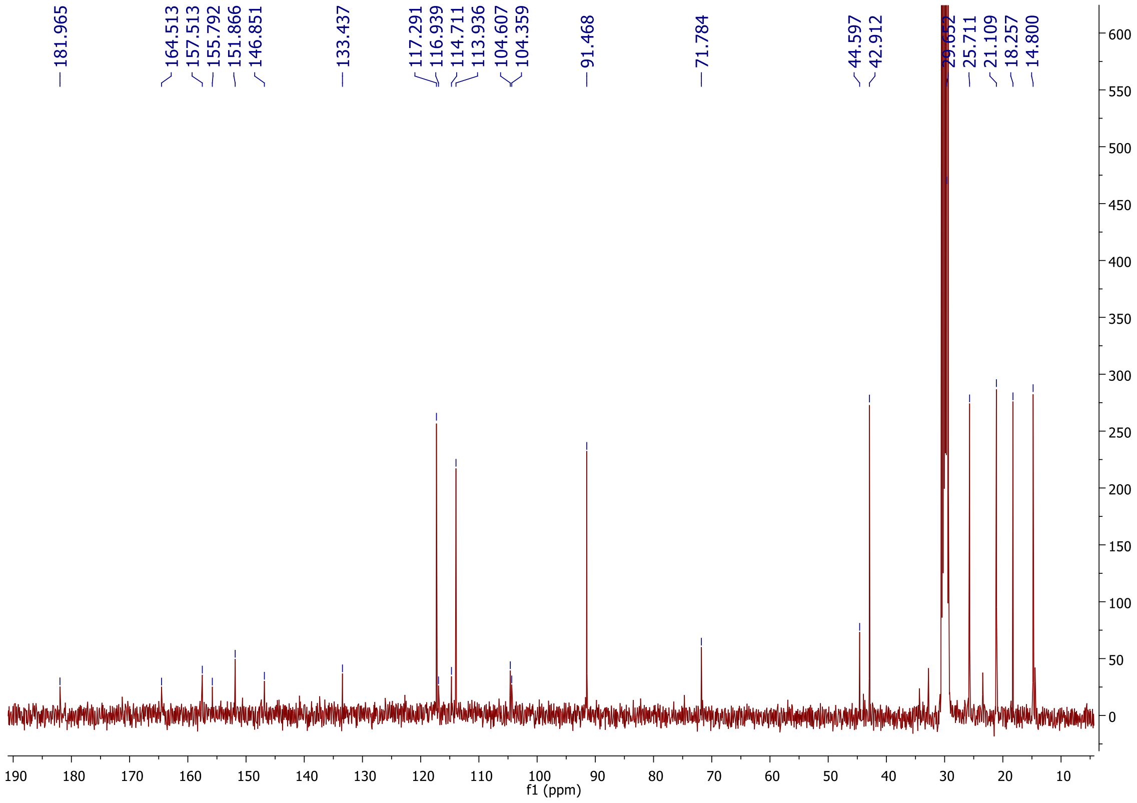

Figure S9. ^{13}C NMR spectrum of gerontoxanthone C hydrate (**4**) in acetone-d_6

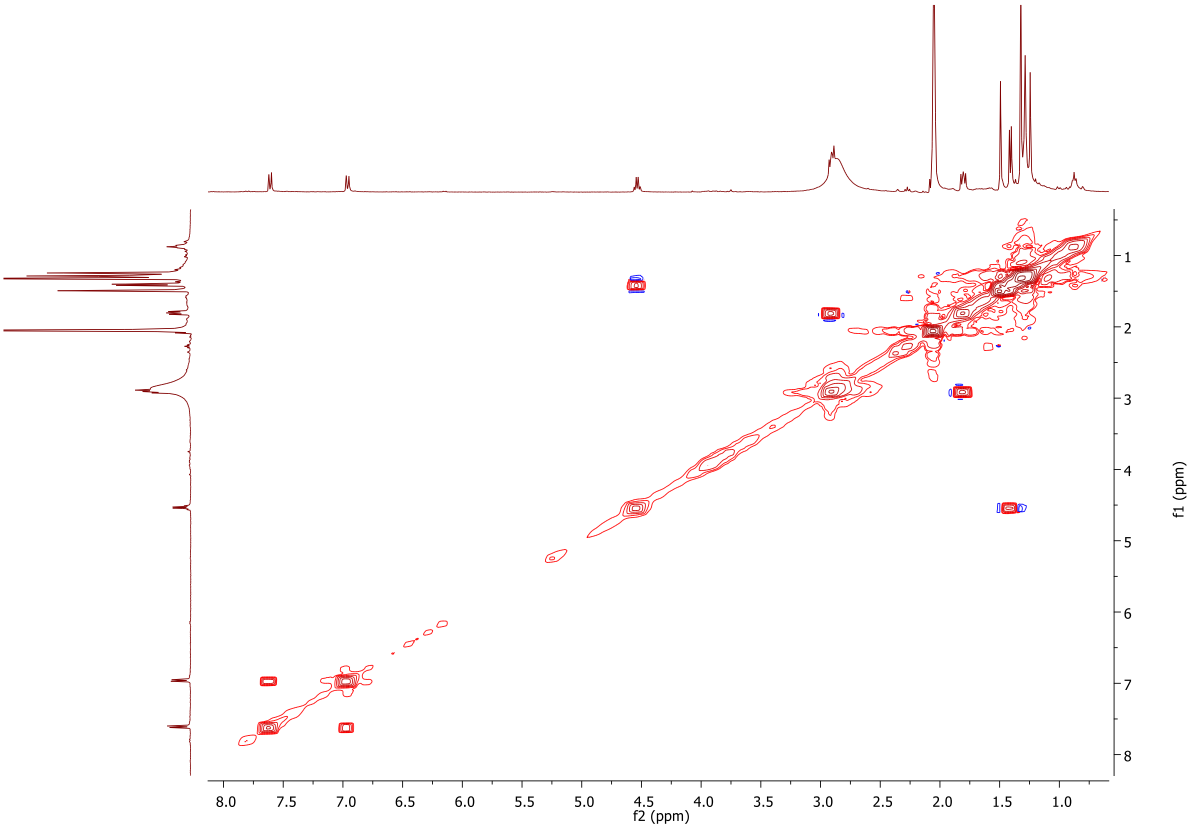

Figure S10. COSY spectrum of gerontoxanthone C hydrate (**4**) in acetone-d_6

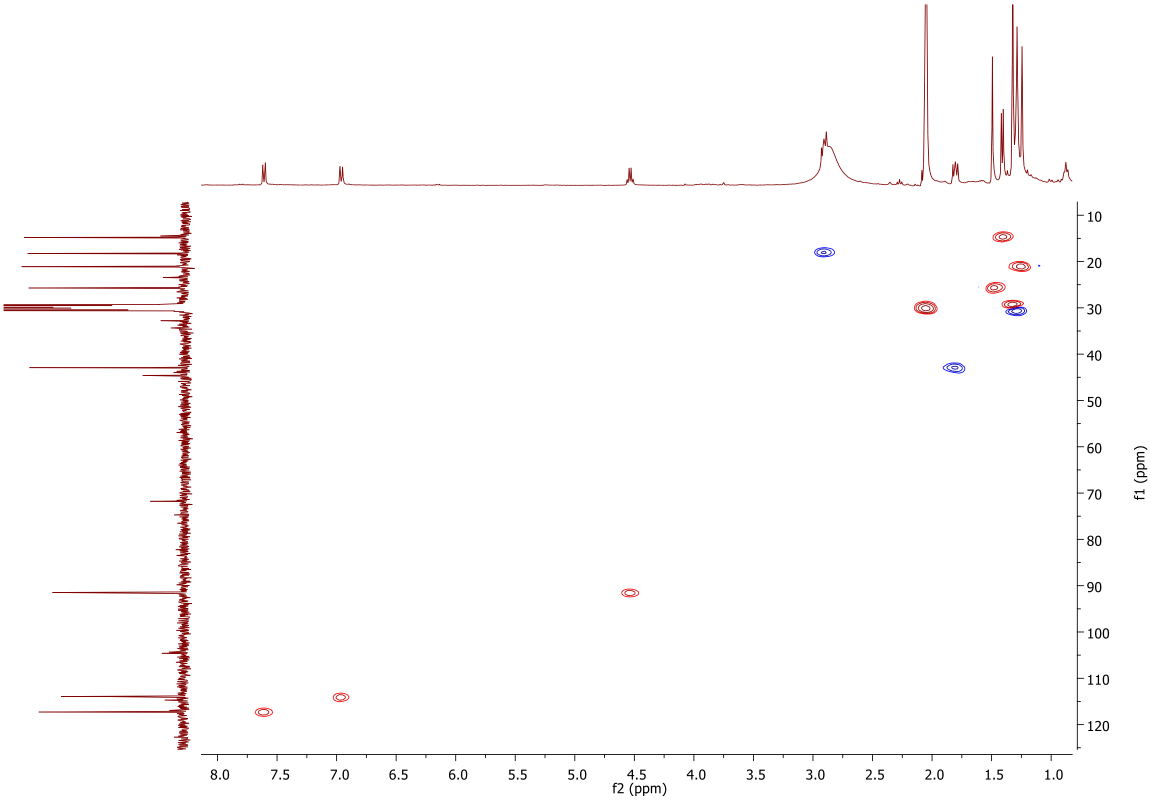

Figure S11. HSQC spectrum of gerontoxanthone C hydrate (**4**) in acetone-d_6

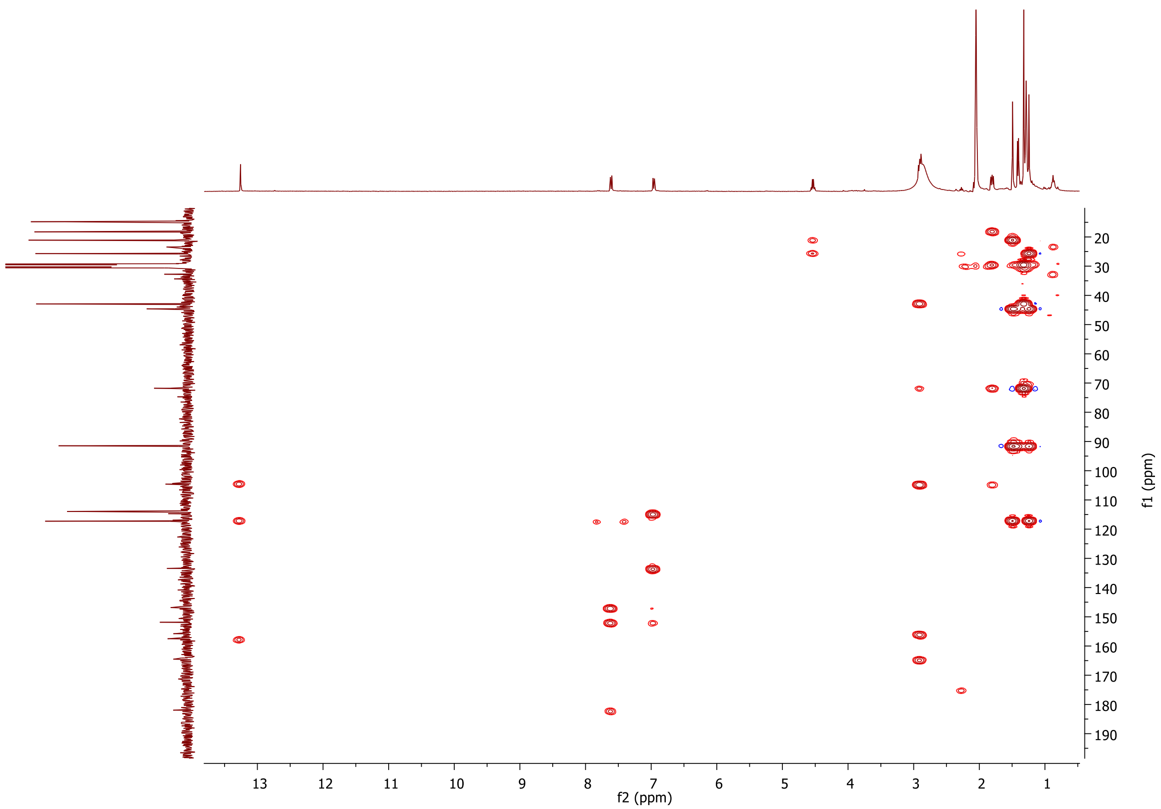

Figure S12. HMBC spectrum of gerontoxanthone C hydrate (**4**) in acetone-d_6

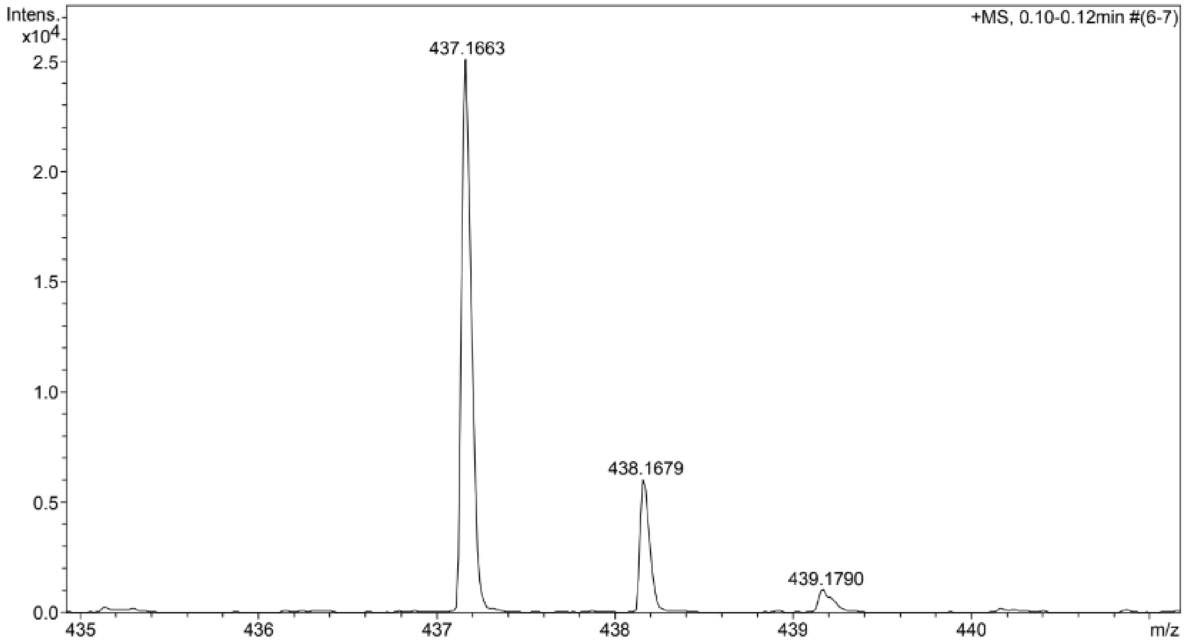

Figure S13. HRESIMS spectrum of gerontoxanthone C hydrate (**4**) in MeOH

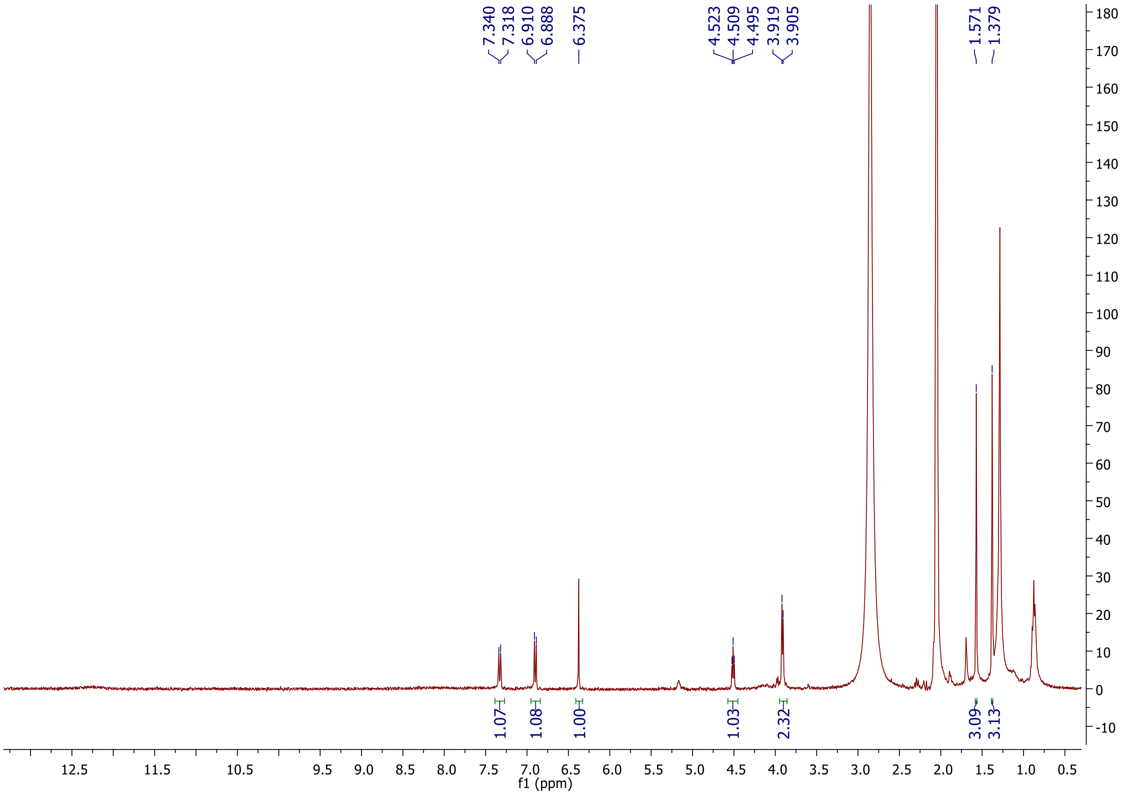

Figure S14. ^{1}H NMR spectrum of 3′-hydroxycalothorexanthone (**5**) in acetone-d_6

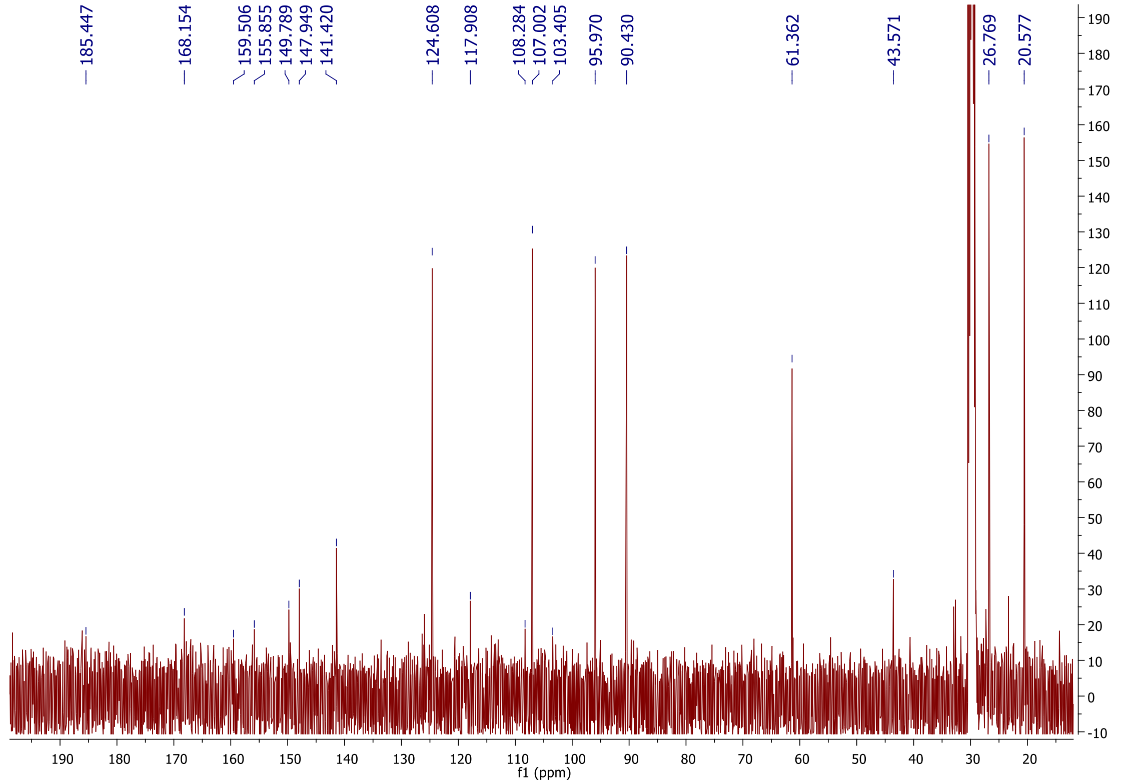

Figure S15. ^{13}C NMR spectrum of 3′-hydroxycalothorexanthone (**5**) in acetone-d_6

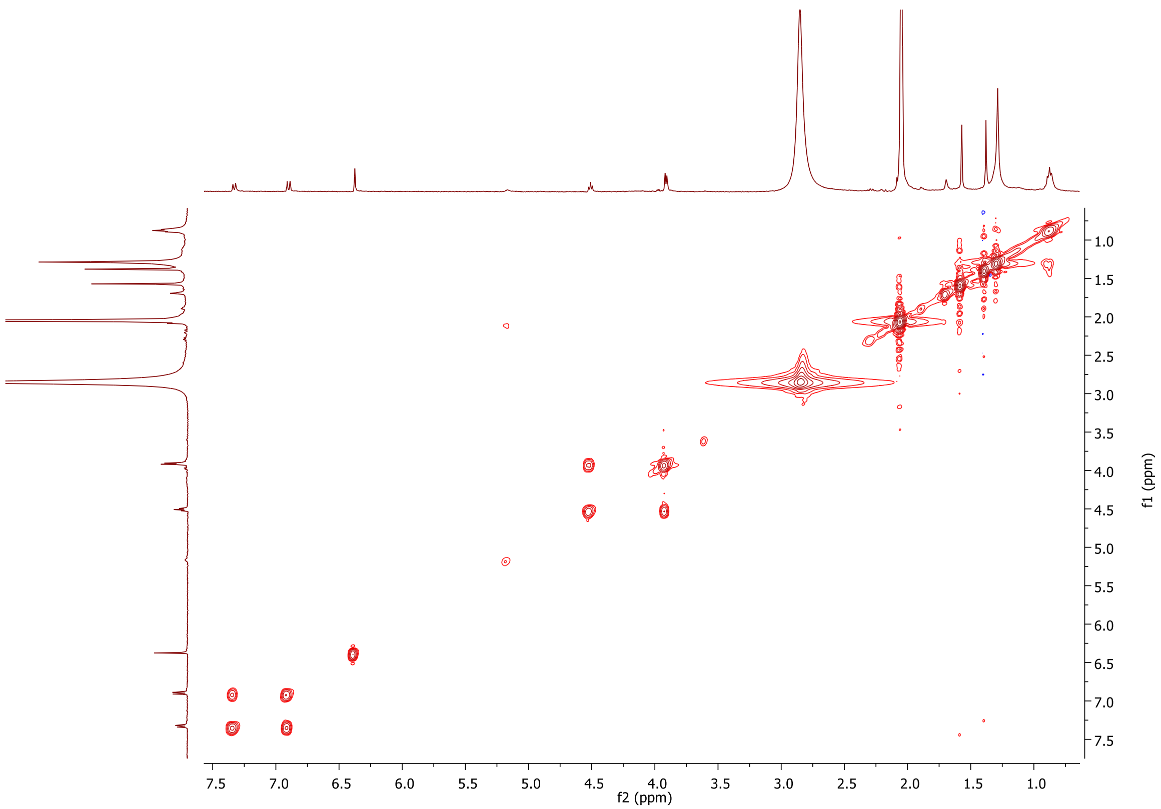

Figure S16. COSY spectrum of 3′-hydroxycalothorexanthone (**5**) in acetone-d_6

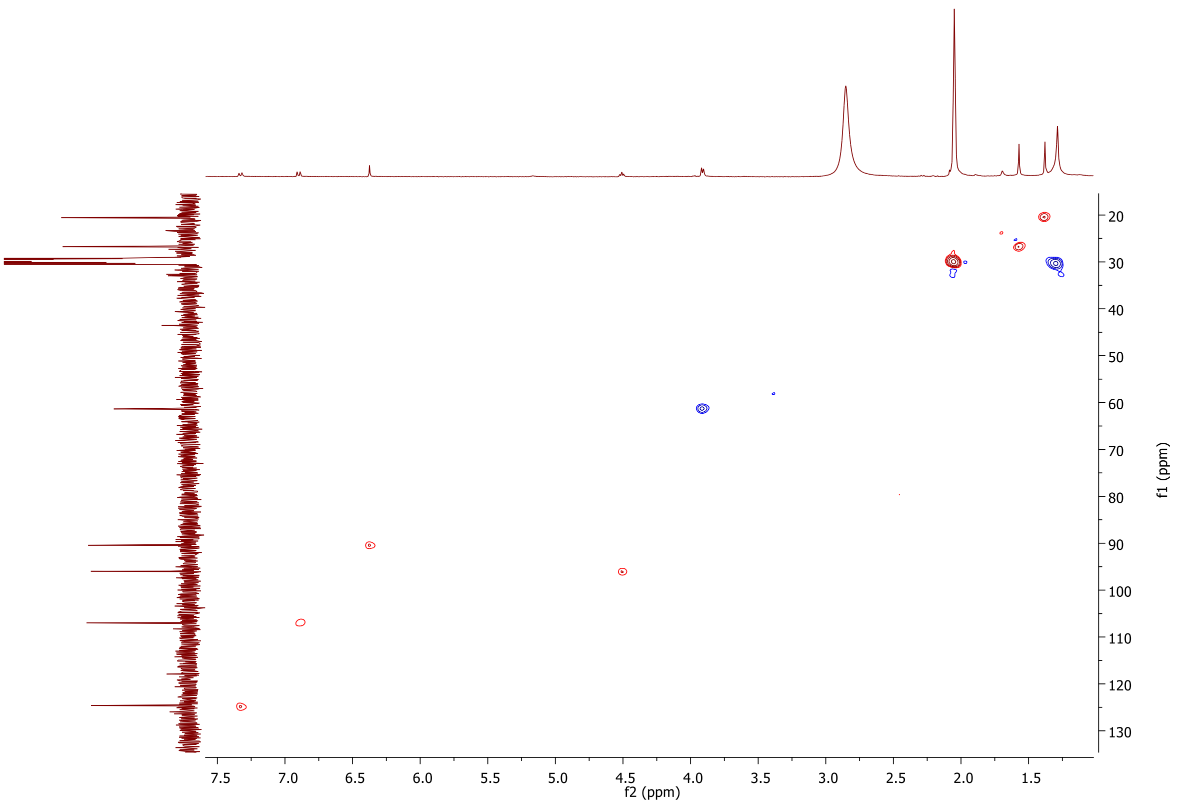

Figure S17. HSQC spectrum of 3′-hydroxycalothorexanthone (**5**) in acetone-d_6

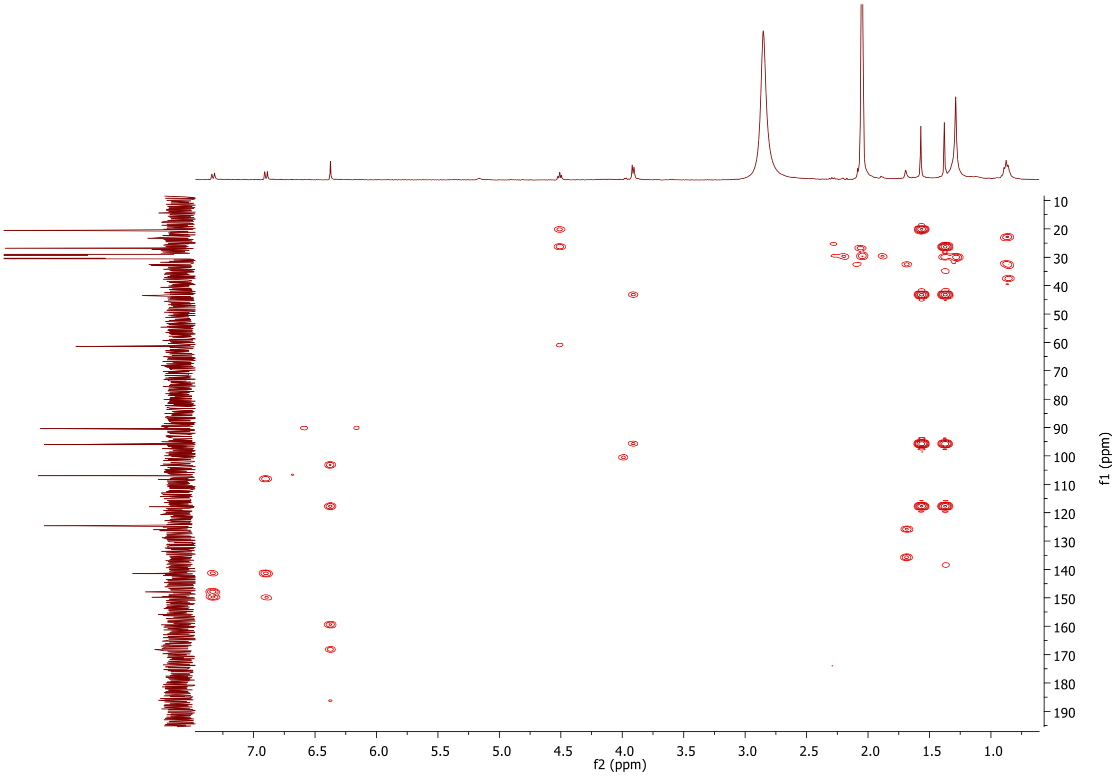

Figure S18. HMBC spectrum of 3′-hydroxycalothorexanthone (**5**) in acetone-d_6

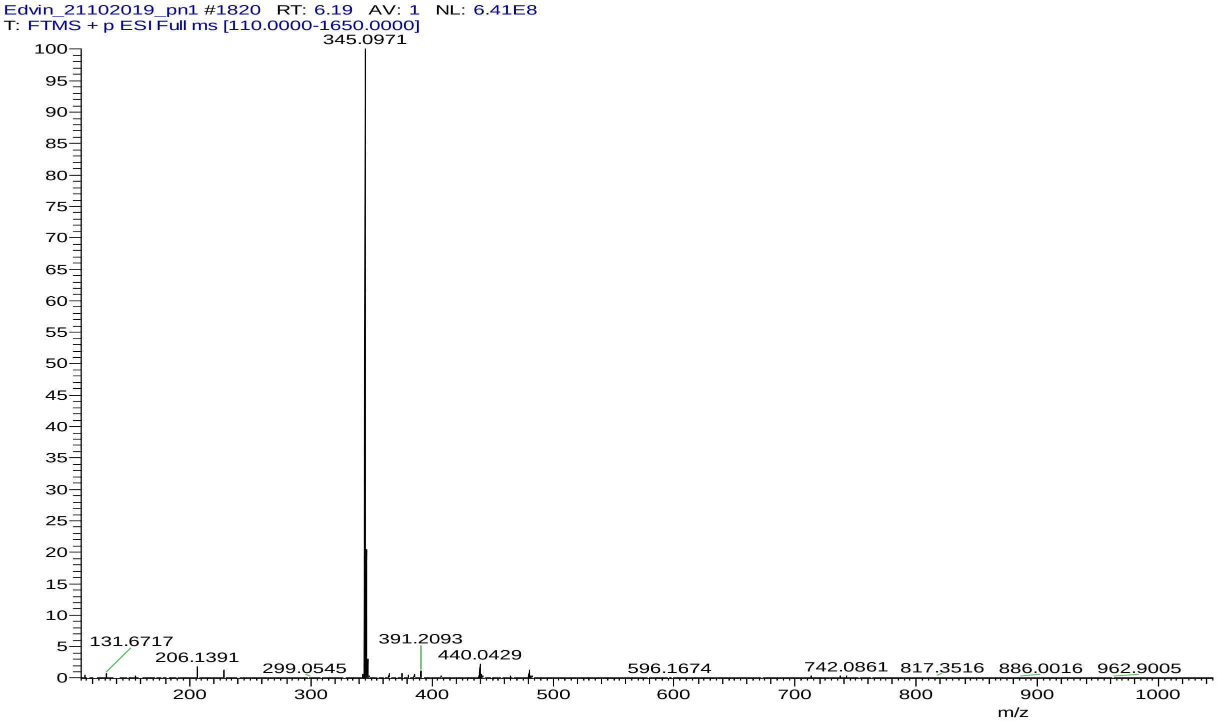

Figure S19. HRESIMS spectrum of 3′-hydroxycalothorexanthone (**5**) in MeOH

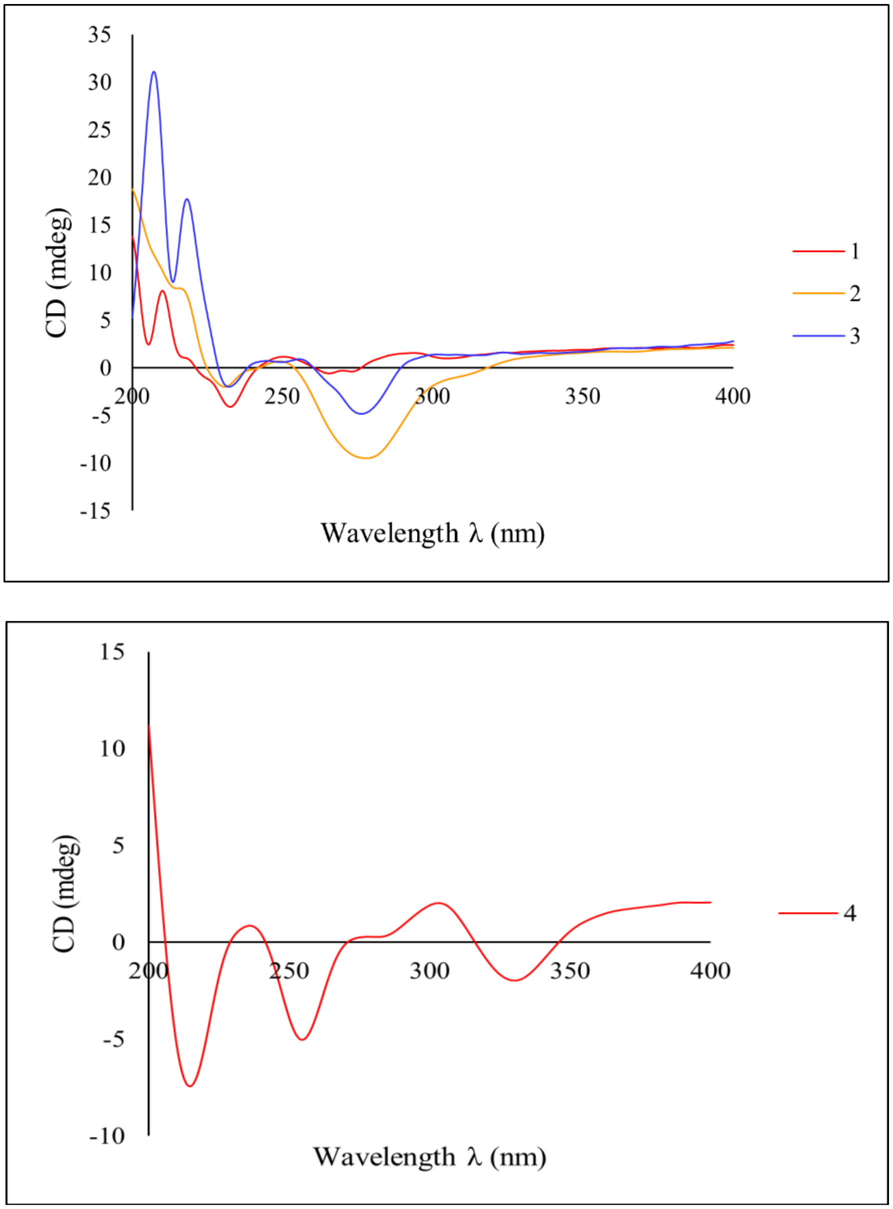

Figure S20. Experimental ECD spectra of **1–4** in MeOH
